# Alcohol myopia and goal commitment

**DOI:** 10.3389/fpsyg.2014.00169

**Published:** 2014-03-04

**Authors:** A. Timur Sevincer, Gabriele Oettingen

**Affiliations:** ^1^Psychology Department, University of HamburgHamburg, Germany; ^2^Psychology Department, New York UniversityNew York, USA

**Keywords:** alcohol intake, alcohol myopia, feasibility, desirability, goal commitment, expectations, incentive value, motivation

## Abstract

According to alcohol myopia theory, acute alcohol consumption leads people to disproportionally focus on the salient rather than the peripheral aspects of a situation. We summarize various studies exploring how myopic processes resulting from acute alcohol intake affect goal commitment. After consuming alcohol student participants felt strongly committed to an important personal goal even though they had low expectations of successfully attaining the goal. However, once intoxicated participants were sober again (i.e., not myopic anymore) they failed to act on their goal commitment. In line with alcohol myopia theory, strong goal commitment as a result of alcohol intake was mediated by intoxicated (vs. sober) participants disproportionally focusing on the desirability rather than the feasibility of their goal. Further supporting alcohol myopia theory, when the *low feasibility* of attaining a particular goal was experimentally made salient (either explicitly or implicitly by subliminal priming), intoxicated participants felt *less* committed than those who consumed a placebo. We discuss these effects of acute alcohol intake in the context of research on the effects of chronic alcohol consumption on goal commitment.

Alcohol intake makes people short-sighted. For example, after having consumed alcohol a layperson, who is thinking about becoming a famous musician, may indulge about giving an acclaimed performance without considering whether she actually has the skill to do so. She may even agree to perform on stage but, once sober again, not engage in the necessary practice and find herself unable to fulfill her commitment. Such a state of alcohol-induced short-sightedness is known as alcohol myopia. The present article reviews research we conducted on the effects of *acute* alcohol consumption on goal-related phenomena, such as commitment to attain important goals, the perceived attractiveness of goals (incentive value), and the subjective likelihood of goal attainment (expectations of success). In addition, we relate our findings to research on the effects of *chronic* alcohol consumption on similar goal-related phenomena.

## ALCOHOL MYOPIA THEORY

According to alcohol myopia theory (Steele and Josephs, 1990), alcohol ingestion leads to a state of short-sightedness by reducing processing capacities (Zeichner and Pihl, 1979; summary by Hull and Slone, 2004). In this state, people disproportionally attend and respond to the most salient information rather than to peripheral information. Information can be salient because particular cues in the environment stand out relative to others (e.g., a red dot surrounded by white dots); it can also be salient because some mental representations are more activated than others (Higgins, 1996). For example, people have a strong need for positive self-regard (Greenwald, 1980). Therefore, when people are asked to evaluate themselves information that supports a positive view likely becomes activated (i.e., salient) in their minds. In contrast to sober people, however, intoxicated people disproportionally focus on the salient positive information. This effect leads them to evaluate themselves overly positive (Banaji and Steele, 1989). In sum, research has investigated alcohol-myopic effects in the domains of aggression, stress, risk-taking, causal inferences, and self-evaluation (summary by Giancola et al., 2010). Here, we focus on the effect of alcohol myopia in the domain of goal commitment.

## GOAL COMMITMENT

Goal commitment can be defined as “attachment to or determination to reach a goal” (Locke et al., 1988, p. 24). The strength of people’s commitment predicts their effort in pursuing a goal and their success in attaining it (Klein et al., 1999). Regarding the determinants of commitment, according to expectancy *x* value theories, the strength of commitment depends on the desirability and feasibility of the goal (Atkinson, 1957; McClelland, 1985; Gollwitzer, 1990). Desirability refers to the subjective attractiveness of the goal (incentive value); feasibility refers to the subjective probabilities of attaining it (expectations of success). Thus, the higher the goal’s desirability and feasibility the stronger the commitment will be. Of importance to the present approach, action-identification theory (Vallacher and Wegner, 1987) suggests that typically a goal’s desirability is more salient to people than its feasibility.

## ALCOHOL MYOPIA AND GOAL COMMITMENT

Our research is based on both alcohol myopia theory (Steele and Josephs, 1990) and action-identification theory (Vallacher and Wegner, 1987). Specifically, we propose that because alcohol intake leads people to disproportionally focus on salient (vs. peripheral) cues and typically the desirability of an important goal is more salient than its feasibility, alcohol intake should lead people to disproportionally focus on a goal’s desirability rather than its feasibility. Focusing on the desirability in turn should lead intoxicated people to strongly commit to their goal even when expectations of attaining it are low.

## EFFECT OF ALCOHOL INTAKE ON GOAL COMMITMENT

In a first study we tested whether acute alcohol intake leads to strong goal commitment even though expectations of goal attainment are low (Sevincer and Oettingen, 2009, Study 1). We asked student participants to name their currently most important goal that pertains to starting or maintaining an interpersonal relationship (e.g., “getting to know someone I like”). We then measured their expectations of success (“How likely do you think it is that you will attain your goal?”). To assure that participants named a goal that is important to them we measured the incentive value of the goal (“How important is it to you that you will attain your goal?”).

Participants then either consumed a placebo or alcohol. Because the sheer belief of having consumed alcohol may affect people’s responses (Hull and Bond, 1986), we used a placebo control condition in which participants were told that they would consume alcohol but in fact they consumed a non-alcoholic beverage. This design allowed us to investigate the pharmacological effect of alcohol ingestion on commitment while participants’ beliefs of having consumed alcohol were held constant (Martin and Sayette, 1993; Sayette et al., 1994). We modeled our procedure for administering the beverages after previous research on the effects of alcohol intake on cognition (e.g., Hull et al., 1983). To enhance the credibility of the placebo, participants in the placebo condition saw their drinks (supposedly vodka-tonic) being poured from a tonic bottle and a bottle labeled vodka that contained decarbonated tonic. They received their drinks at a dilution that did not allow them to reliably detect whether the drink contained alcohol or not (Marlatt et al., 1973). Further, they were subjected to a breathalyzer test in which they saw their bogus blood alcohol content (BAC) displayed. The bogus BAC was a random value close to the mean BAC in the alcohol condition (0.04%). We assessed the dependent variable, goal commitment, by asking: “How disappointed would you feel if you did not attain your goal?” As strongly committed people are typically highly disappointed when failing to attain their goal, the degree of anticipated disappointment in case of failure reliably indicates commitment (Wicklund and Gollwitzer, 1982; Oettingen et al., 2001). Finally, in all studies reported here, we checked the effectiveness of the placebo manipulation by asking participants to report how much alcohol they thought they had consumed. Across studies, at least 94% of the participants in the placebo conditions reported having consumed alcohol. Those who reported having consumed no alcohol were discarded from the analyses.

**FIGURE 1:**
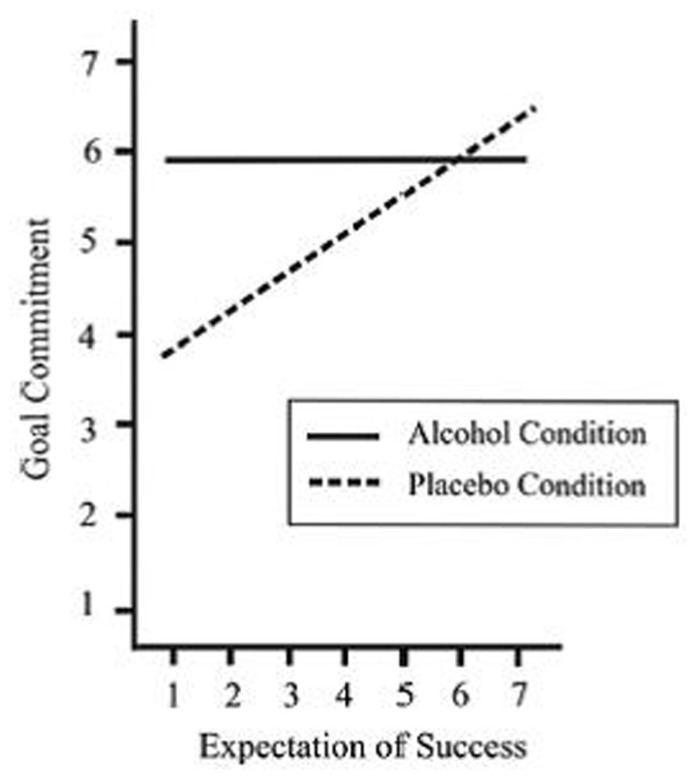
**Regression lines depict the link between expectations of success and goal commitment as a function of condition (alcohol vs. placebo).** From Sevincer and Oettingen (2009, Study 1).

Participants who consumed a placebo felt strongly committed when they had high expectations, but only weakly committed when they had low expectations. Participants who consumed alcohol, in contrast, felt strongly committed regardless of whether they had high or low expectations. Moreover, in light of low expectations, participants who consumed alcohol (vs. a placebo) felt more committed (**Figure 1**).

In line with alcohol myopia theory and action-identification theory alcohol intake led to strong commitment regardless of whether expectations were high or low. As commitment strongly predicts goal-directed action over time, we investigated whether participants would go ahead and act on their strong commitment once they were sober again. We hypothesized that when the effects of the alcohol have worn off, participants would consider their low expectations again. Thus, once sober, participants with low expectations should refrain from goal-directed actions.

## GOAL-DIRECTED ACTION OVER TIME

The second study reported here (Sevincer and Oettingen, 2009, Study 2) followed the same basic procedure as the first one. Participants named their currently most important interpersonal goal and indicated respective expectations and incentive value. They then consumed either a placebo or alcohol (mean BAC: 0.05%), and self-reported their commitment on three items (e.g., “How determined are you to attain your goal?”). To examine whether intoxicated participants’ strong commitment would translate into goal-directed action over time, 3 weeks after the experiment we sent participants a follow-up questionnaire. We asked them to list all actions they had undertaken to attain their goal since taking part in the experiment (they listed e.g., “I apologized to my friend”).

We replicated the results of the first study: Participants in the placebo condition only felt strongly committed when expectations were high; those in the alcohol condition felt strongly committed regardless of whether they had high or low expectations. Over time, as predicted, goal-directed action did not differ between the placebo and the alcohol condition. The number of actions performed after 3 weeks depended on participants’ expectations, in both the placebo and the alcohol condition. In the placebo condition but not in the alcohol condition commitment mediated the effect of expectations on goal-directed action over time. These findings suggest that alcohol ingestion breeds empty goal commitment: Intoxicated participants felt strongly committed even though they had only low expectations, and once sober, they did not follow through.

## FOCUS ON DESIRABILITY vs. FEASIBILITY AS A MEDIATOR

Thus far, we showed that alcohol ingestion leads to strong goal commitment regardless of low expectations. We did not yet test, however, whether this effect occurs because intoxicated people indeed disproportionally focus on desirability rather than feasibility. In our next study (Sevincer and Oettingen, 2013a) participants again named a currently important goal (this time related to acquiring a desired future identity, e.g., “becoming a lawyer”). They also indicated their respective expectations and incentive value. Then they consumed either a placebo or alcohol (mean BAC: 0.07%). To explore whether participants who consumed alcohol (vs. a placebo) would disproportionally focus on desirability (vs. feasibility) we asked them to freely write about their goal. We content analyzed the written elaborations according to the extent to which they contained statements about desirability and statements about feasibility. Specifically, two independent raters, unaware of the hypotheses, first segmented participants’ elaborations into single statements (Sevincer and Oettingen, 2013b). They then coded each statement according to whether it pertained to desirability (e.g., “I would earn much money”), feasibility (e.g., “it is difficult to pass the final exam”), or neither (e.g., “the semester starts next week”). Interrater agreement was 88%. Finally, participants self-reported their commitment on the same three items as used in Sevincer and Oettingen (2009, Study 2).

Participants who consumed alcohol (vs. a placebo) wrote more about desirability and less about feasibility. Moreover, they felt strongly committed despite low expectations; this effect was mediated by the number of statements related to desirability (vs. feasibility). The findings suggest that intoxicated more than sober people disproportionally focus on desirability rather than feasibility and thus feel strongly committed to their goals.

## COMMITMENT WHEN LOW EXPECTATIONS ARE SALIENT

According to alcohol myopia theory, alcohol intake may either increase or decrease people’s responses depending on which information is most salient. For example, intoxicated (vs. sober) participants reacted more aggressively when a provocative cue (receiving a shock) was salient, but reacted less aggressively when a cue distracting from the provocation (engaging in a task) was salient (Giancola and Corman, 2007). Similarly, intoxicated (vs. sober) participants became more anxious when a stress-evoking cue (giving a speech) was salient, but became less anxious when a cue distracting from the stressor (rating art slides) was salient (Josephs and Steele, 1990). Intoxicated (vs. sober) participants were more willing to engage in unprotected sexual intercourse when impelling cues (having a seemingly trustworthy partner) were salient, but were less willing when inhibiting cues (being reminded of not having a condom) were salient (MacDonald et al., 2000). Alcohol intake also altered causal inferences, leading to exaggeration of either dispositional or situational causes for behavior, depending on which cues were more salient (Herzog, 1999). Thus, an even more stringent test of whether alcohol myopia is a causal mechanism by which alcohol ingestion leads people to feel strongly committed to a goal of low feasibility, would be to make the goal’s low feasibility more salient than its desirability. Then, intoxicated (vs. sober) people should feel *less *committed. We tested this hypothesis in two studies (Sevincer et al., 2012).

Previous research demonstrated alcohol-myopic effects by manipulating the salience of cues in an explicit way. Cues were made salient, for example by highlighting information in a questionnaire (MacDonald et al., 2000), pointing out information during a conversation (MacDonald et al., 1995), or presenting words in red script (Curtin et al., 2001). However, mental representations can also become implicitly activated (i.e., made salient). In subliminal priming, a particular mental representation is activated when a stimulus is presented below the threshold of conscious perception. The activated representation in turn influences people’s responses (Higgins, 1996; Bargh and Chartrand, 2000). We therefore tested (a) whether alcohol intake leads people to feel less rather than more committed when low expectations are made salient and (b) whether this effect occurs not only when low expectations are explicitly activated (Study 1), but also when they are implicitly activated (Study 2).

## EXPLICIT ACTIVATION OF LOW EXPECTATIONS

To investigate whether explicitly activating low expectations would lead participants who consumed alcohol (vs. a placebo) to feel less committed, we had participants name an identity goal, for which they had low expectations. Specifically, we asked: “Please name a personal goal directed at acquiring a specific future identity that is important to you but that you think you are unlikely to attain” (they named e.g., “becoming a professional soccer player”). They also indicated their expectations and incentive value. Thereafter, they consumed either a placebo or alcohol (mean BAC: 0.07%). Then participants’ low expectations were either not activated or explicitly activated. To manipulate the explicit activation of low expectations (i.e., activation absent vs. present), we followed a procedure by MacDonald et al. (2000). Specifically, we embedded the manipulation in the assessment of the dependent variable, which was self-reported commitment as measured by five items. In the activation absent condition, we asked for example: “How disappointed would you be if you did not attain your goal.” In the activation present condition, we added the subordinate clause “**that you think you are unlikely to attain” **in bold type to every item (e.g., “How disappointed would you be if you did not attain your goal **that you think you are unlikely to attain**”).

As predicted, when low expectations were not activated, commitment did not differ between the placebo and the alcohol condition. In contrast, when low expectations were explicitly activated participants who consumed alcohol (vs. a placebo) felt less committed. Thus, explicitly making low expectations salient led intoxicated participants to feel less committed than those who consumed a placebo.

## IMPLICIT ACTIVATION OF LOW EXPECTATIONS

Would the implicit activation of low expectations be enough to produce lower goal commitment in intoxicated participants? Participants named an interpersonal goal that was important to them, but for which they had low expectations. After indicating their expectations and incentive value they either consumed a placebo or alcohol (mean BAC: 0.07%). Then, their low expectations were either not activated or implicitly activated. To implicitly activate low expectations we used a subliminal priming procedure (Bargh and Chartrand, 2000). Whereas in the activation absent condition participants were subliminally presented with a neutral control stimulus (a letter string: “*xxxxxxxxxxx*”), in the activation present condition they were subliminally presented with words related to low expectations (“*unattainable*,” “*unrealizable*”). Finally, participants self-reported their commitment on the same five items as used in the activation absent condition in Study 1.

As predicted, when participants were subliminally primed with a neutral control stimulus, commitment did not differ between the placebo and the alcohol condition. In contrast, when subliminally primed with low expectations, participants who consumed alcohol (vs. a placebo) felt less committed. This finding suggests that alcohol-myopic effects can be triggered by stimuli that people are not even aware of, just like by stimuli that people consciously process.

## CHRONIC ALCOHOL CONSUMPTION AND GOAL-RELATED PHENOMENA

The aim of the present article is to illuminate how acute alcohol consumption can affect motivational constructs, as identified by traditional expectancy *x* value theories (e.g., goal commitment, expectations of success, incentive value; Atkinson, 1957; Klinger, 1975; McClelland, 1985). Relatedly, the motivational model of alcohol use by Cox and Klinger (1988, 2011a) employs an expectancy *x* value framework to explore people’s chronic alcohol consumption. According to the model, people’s decision to drink or not to drink alcohol in a particular situation is determined by whether they expect that drinking will result in attractive outcomes (e.g., increased positive affect or decreased negative affect; Cooper et al., 1995). The decision to drink alcohol, however, competes with other goals. That is, people reduce their drinking if they expect that they can attain attractive outcomes by acting on alternative goals (e.g., establishing satisfying relationships). In support of this contention, students who used to drink heavily, but then turned to alternative goals, reduced their drinking (Cox et al., 2002). Similarly, compared to people who were no heavy drinkers, heavy drinkers (i.e., those diagnosed with alcoholism) reported to be less engaged in alternative goals and had relatively low expectations of attaining alternative goals (Man et al., 1998).

Our results add to these findings. For example, they suggest that acute alcohol consumption may temporarily increase people’s engagement in alternative but unfeasible goals. Thus, intoxicated people may feel momentarily excitied about alternatives to drinking. When sober, however, as people fail to act on the alternative goals, they may feel disappointed and frustrated. This effect may on the long run, undermine people’s engagement in alternative goals. Clinical interventions (e.g., motivational counseling; Cox and Klinger, 2011b) may thus focus on cautioning people who chronically consume alcohol from becoming overly committed to important goals while intoxicated. Finally, based on the finding by Man et al. (1998) that participants who chronically (vs. non-chronically) consume alcohol report lower expectations of attaining important personal goals, our research suggests that consuming alcohol may be particularly attractive for chronic alcohol users, because alcohol distracts them from their low prospects to reach alternative life pursuits.

## SUMMARY

Consuming alcohol may lead to strong goal commitment by making people disproportionally focus on the desirability rather than feasibility of important goals. However, once sober, people do not act on their strong commitment. Of importance though, in a situation where low expectations are activated (i.e., made salient), either explicitly or implicitly, alcohol intake leads to weak commitment. Looking back to the example at the beginning of this article, the aspiring musician may feel strongly committed to performing on stage *because* the alcohol myopia made her focus on the high desirability of giving an excellent performance. Once sober and not myopic anymore, however, she is unlikely to follow through on her commitment. Reminding her – explicitly or implicitly – of the low feasibility of giving an excellent performance should have made her feel even less committed while intoxicated than when sober. The reported findings complement research on chronic alcohol consumption and goal-related phenomena (goal commitment, expectations of success, incentive value; Cox and Klinger, 2011a) by illuminating how acute alcohol consumption affects these phenomena.

## Conflict of Interest Statement

The authors declare that the research was conducted in the absence of any commercial or financial relationships that could be construed as a potential conflict of interest.
